# Can frailty predict complications and mortality in patients undergoing total knee arthroplasty? A systematic review and meta-analysis

**DOI:** 10.12669/pjms.42.8.16131

**Published:** 2026-08

**Authors:** Bo Jin, Jianyue Wang, Yungen Hu, Weifan Ren

**Affiliations:** 1 Bo Jin, Department of Joint, Xiaoshan District Hospital of Traditional Chinese Medicine, Hangzhou, Zhejiang Province 311200, P.R. China; 2 Jianyue Wang, Department of Joint, Xiaoshan District Hospital of Traditional Chinese Medicine, Hangzhou, Zhejiang Province 311200, P.R. China; 3 Yungen Hu, Department of Joint, Xiaoshan District Hospital of Traditional Chinese Medicine, Hangzhou, Zhejiang Province 311200, P.R. China; 4 Weifan Ren, Department of Joint, Xiaoshan District Hospital of Traditional Chinese Medicine, Hangzhou, Zhejiang Province 311200, P.R. China

**Keywords:** Frailty, Total Knee Arthroplasty, Knee Replacement, Complications, Mortality

## Abstract

**Objective:**

To evaluate the value of frailty in predicting postoperative complications and mortality in patients undergoing total knee arthroplasty (TKA).

**Methodology:**

PubMed, Scopus, EMBASE and CENTRAL databases were screened up to 20^th^ November 2025 for observational and randomized studies evaluating postoperative outcomes in frail TKA patients. Data on postoperative complications, readmission and reoperation rates, length of hospital stay and mortality were extracted. A random-effects model was used to calculate pooled risk ratios (RRs) and odds ratios (ORs) with 95% confidence intervals (CIs).

**Results:**

Sixteen studies with over 14 million patients were included. Frail patients had a significantly higher risk of 30-day mortality with RR 4.40 (95% CI: 2.18-8.91; p < 0.00001); overall postoperative complications with RR 1.85 (95% CI: 1.35-2.52; p= 0.0001); 30-day readmission with RR: 2.24 (95% CI: 1.74-2.87; p < 0.00001) and 30-day reoperation with RR: 1.81 (95% CI: 1.48-2.22; p < 0.0001). Frailty was also linked to an increased risk of surgical site infections with RR 1.56 ( 95% CI: 0.86–2.83) and prolonged hospital stay with mean difference MD 0.86 days (95% CI: 0.55–1.16; p<0.00001). Individual complications, such as myocardial infarction, renal complications and pulmonary embolism, were consistently more frequent in frail patients. Heterogeneity was moderate to high in some outcomes, attributed to differences in frailty assessment tools and study designs.

**Conclusion:**

Within the limitations, frailty is a strong, independent predictor of increased postoperative complications, mortality and prolonged hospital stays in patients undergoing TKA. Incorporating frailty assessments into preoperative evaluations can enhance risk stratification and guide interventions to improve perioperative care in this vulnerable population.

**Prospero Registration Number:** CRD420251035972.

## INTRODUCTION

Total knee arthroplasty (TKA) is commonly used to alleviate chronic joint pain and enhance mobility in patients with advanced degenerative knee conditions, such as osteoarthritis, rheumatoid arthritis and post-traumatic arthritis.^1^,^2^ With the aging of the global population, the volume of TKA procedures has surged, particularly among older adults.^3^,^4^ While this approach offers substantial benefits in terms of functional restoration and health-related quality of life, patients’ physiological reserve, most notably characterized by the construct of frailty, is considered a key determinant of variability in postoperative outcomes of TKA.^5^,^6^

Frailty is characterized by a decline in physiological systems, reduced strength and endurance and impaired homeostatic mechanisms, which increase vulnerability to stressors such as surgery, predisposing patients to a spectrum of adverse outcomes.^7^,^8^ Elderly frail patients undergoing TKA are particularly susceptible to postoperative complications, including infections, impaired wound healing, thromboembolic events, acute kidney injury and cardiac events,^9^,^10^ which increases the likelihood of intensive care admissions, prolonged hospital stays, unplanned readmissions and discharge to assisted living facilities or rehabilitation centers.

Beyond immediate perioperative risks, frailty also significantly impairs long-term functional recovery and quality of patients undergoing TKA.^11^,^12^ Frail patients often experience slower gains in mobility, which makes them less able to participate in rehabilitation and increases the risk of social dependency and report lower rates of satisfaction with surgical outcomes.^13^ Furthermore, frailty is associated with higher mortality rates in the short- and long-term periods after TKA due to decreased resilience of frail patients to surgical stress and underlying systemic vulnerabilities.^14^,^15^

Emerging evidence suggests that frailty is strongly associated with an increased risk of postoperative infectious and thromboembolic complications following total hip and knee arthroplasty. A recent meta-analysis by Wu et al.^16^ demonstrated that frail patients had a significantly higher risk of surgical site infections, particularly deep surgical site infections and periprosthetic joint infections, after THA/TKA. Similarly, Lan et al.^17^ reported that preoperative frailty significantly increased the risk of venous thromboembolism, including deep vein thrombosis and pulmonary embolism, in arthroplasty patients. These findings highlight the biological vulnerability of frail individuals, likely driven by impaired immune function, chronic inflammation, reduced mobility and diminished physiological reserve, further supporting the importance of frailty assessment in predicting postoperative outcomes in TKA patients.

While studies confirm the prognostic value of frailty in TKA, there are still no attempts to systematically review and evaluate the extent to which frailty influences postoperative complications and mortality in this population. The present study aimed to critically appraise and summarize the available research on the predictive value of frailty in determining postoperative complications and mortality among patients undergoing TKA.

## METHODOLOGY

The Preferred Reporting Items for Systematic Reviews and Meta-Analyses (PRISMA) 2020 guidelines^18^ were followed. The protocol was preregistered with the International Prospective Register of Systematic Reviews (PROSPERO), registration number CRD420251035972.

### Research question:

Can Frailty Predict Complications and Mortality in Patients Undergoing Total Knee Arthroplasty?

The study addressed the above research question, developed using the PEO (Population, Exposure, Outcome) framework:

- Population (P): Adults undergoing total knee arthroplasty.
- Exposure (E): Frailty (as assessed by validated tools).
- Outcome (O): Postoperative complications and mortality.

### Search strategy:

PubMed, Scopus, EMBASE and the Cochrane Central Register of Controlled Trials (CENTRAL) databases were searched for studies up to November 2025 using both Medical Subject Headings (MeSH) and free-text keywords related to “knee arthroplasty,” “frailty,” “complications,” and “mortality.” Boolean operators (“AND”, “OR”) were used to construct search strategies tailored to each database. The search string for PubMED is as follows: (“Arthroplasty, Replacement, Knee”[Mesh] OR “knee arthroplasty” OR “total knee arthroplasty” OR “knee replacement” OR “total knee replacement” OR “TKA”) AND (“Frailty”[Mesh] OR “frailty syndrome” OR “frailty index” OR “frail elderly” OR “frailty phenotype” OR “frail patient*” OR “clinical frailty scale” OR “frailty scale” OR “frailty score” OR “frail*”) AND (“Postoperative Complications”[Mesh] OR “Surgical Wound Infection”[Mesh] OR “Intraoperative Complications”[Mesh] OR “Postoperative Period”[Mesh] OR “Hospital Mortality”[Mesh] OR “mortality”[Mesh] OR “complication*” OR “adverse outcome*” OR “postoperative complication*” OR “surgical complication*” OR “30-day mortality” OR “hospital mortality” OR “in-hospital mortality” OR “death”). The search was last performed on 20^th^ November 2025. Additionally, references of included studies and relevant review articles were manually searched (snowballing). No language restrictions were applied to the search.

Reference management software (EndNote v17.0, Clarivate Analytics, USA) was used to import the retrieved studies, which were then screened for eligibility. Duplicate entries across databases were identified and removed using the citation manager’s automated tools, followed by manual verification to ensure accuracy. Two reviewers independently performed titles and abstracts screening. Full-text screening of potentially relevant studies was then conducted and any disagreements were resolved through discussion or consultation with a third reviewer.

### Study selection:

Titles and abstracts were first screened by the two independent reviewers for eligibility. Studies meeting the requirements were advanced to the second phase of the full-text screening.

### Inclusion Criteria:

- Studies involving adult patients (≥18 years) undergoing primary or revision TKA.
- Studies assessing frailty status (using validated frailty tools such as Fried criteria, Clinical Frailty Scale, Frailty Index, etc).
- Studies reporting postoperative complications (e.g., infection, thromboembolism, cardiac events, delayed wound healing), mortality, or other adverse outcomes based on frailty status.
- Observational studies (prospective or retrospective cohort, case-control studies) and randomized controlled trials.

### Exclusion Criteria:

- Studies not reporting relevant outcome data stratified by frailty status.
- Studies focusing on other types of joint replacements (e.g., hip arthroplasty) without separate data for TKA.

### Data extraction:

Data was independently extracted by the two independent reviewers (B.J. and J.W.) using a pre-tested and standardized form. Study characteristics (author, year, country, study design), population details (sample size, age, sex distribution), frailty assessment tools used, outcome definitions, duration of follow-up and reported effect estimates (crude and adjusted odds ratios and 95% confidence intervals) were collected. Any inconsistencies were settled through discussion or consultation with a third reviewer.

### Risk of bias assessment:

The methodological quality of included studies was assessed by the two reviewers independently. The Newcastle-Ottawa Scale (NOS) was used to evaluate selection, comparability and outcome domains of the cohort and case-control studies. NOS evaluates three major domains: selection of study groups, comparability of groups and ascertainment of the outcome, with a total possible score of Nine.

For randomized controlled trials, the Cochrane Risk of Bias 2.0 tool was used to categorize studies as low, moderate, or high risk of bias. A third reviewer adjudicated any discrepancies.

### Data synthesis and Statistical analysis:

All quantitative analyses were performed using Review Manager (RevMan 5.4version, Cochrane Collaboration, UK). Effect estimates (Odds ratios, Risk Ratios for dichotomous data and Mean difference for continuous data) were pooled using a random-effects model to account for anticipated clinical heterogeneity. Separate analyses were conducted for crude and adjusted data wherever available. The I² statistic assessed statistical heterogeneity. I² of 25%, 50% and 75% indicated low, moderate and high heterogeneity, respectively. Subgroup analyses were planned based on the individual complications. The certainty of evidence was assessed using GRADE.

## RESULTS

**Fig.1:**
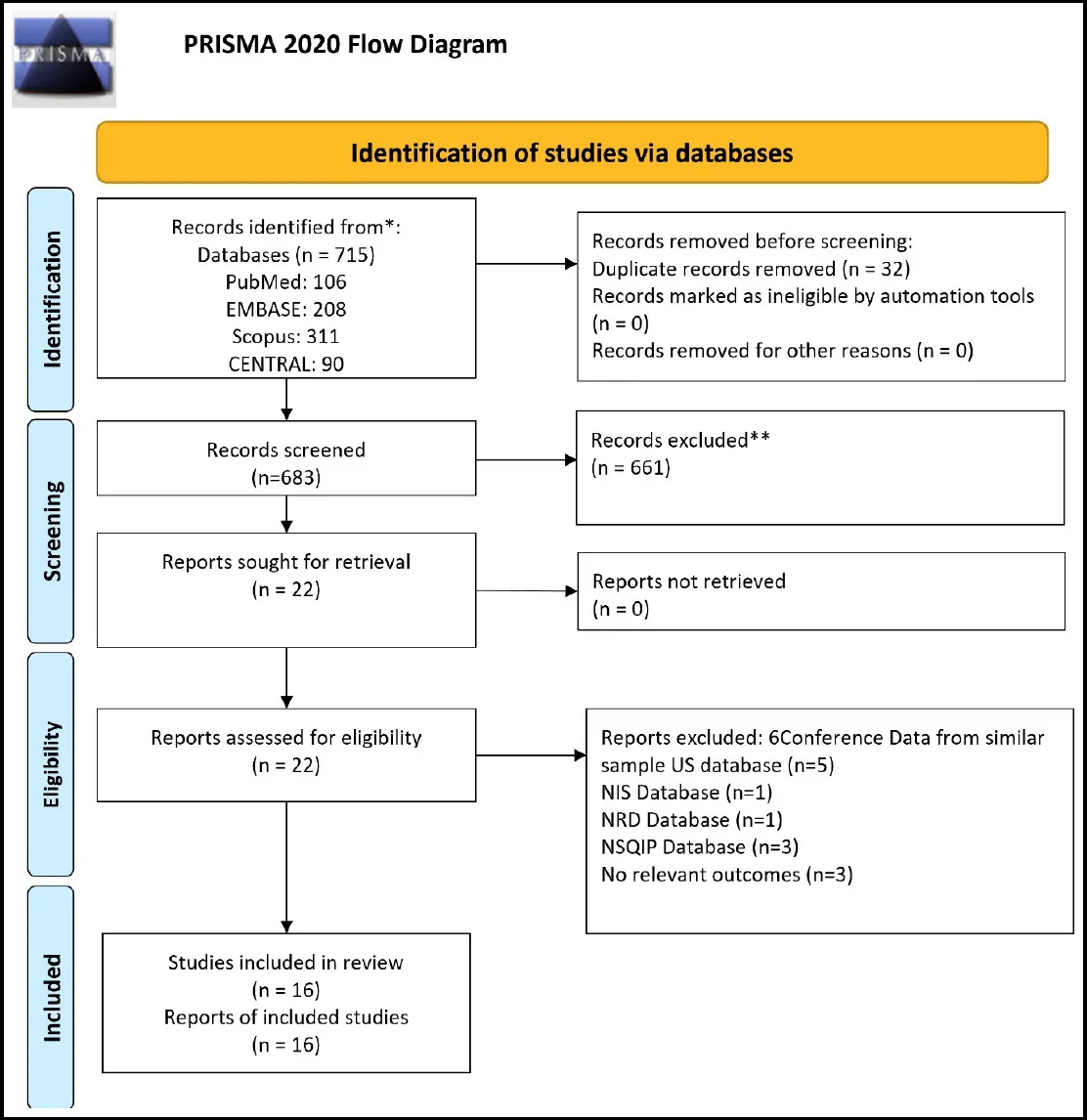
Study Selection Flow Chart.

The detailed study screening shown in Fig.1, the search identified 715 records in PubMed (n = 106), EMBASE (n = 208), Scopus (n = 311) and CENTRAL (n = 90) databases. After deduplication, 683 unique records were screened based on their titles and abstracts and 661 records that did not meet the inclusion criteria were eliminated. Of the remaining 22 full-text articles that were assessed for eligibility, six were excluded due to overlapping data from using similar U.S. databases (n = 5), use of the same national databases, such as NIS,^19^ NRD,^20^ and NSQIP,^21^ or for not reporting relevant outcomes related to frailty or postoperative complications.^22^ Ultimately, 16 studies^23^–^38^ were included in the final qualitative synthesis and pooled analysis (Table-I).

**Table-I:** Characteristics of Included Studies.

Author Year Country Study Period	Study Design	Database/ Study Setting	Sample Size	Male	Female	Age in years	TKA N (%)	Frail N (%)	Frailty Index	Follow-up	Outcomes Assessed	Adjusted For
O’Connor et al. 2025 USA 2015 - 2023	Retrospective Cohort Study	Hospital’s electronic medical record system (EMRs)	4,082	1688	2394	68 ± 9.4 (Frail) 67 ± 9.1 (Non-Frail)	4,082	42.30%	Hospital frailty risk score (HFRS)	90 days	Length of Stay, Revision of TKA, 90-Day Readmission Rates	Age, sex, BMI, race (%), ethnicity (%)
Wall et al. 2025 Australia Feb 2019 – Feb 2020	Retrospective observational study	EMRs and institutional database	622	NR	184 (56.1%)	67.5 ± 9.8	328 (100%)	NR	Clinical Frailty Scale (CFS)	Six-weeks	Hospital LOS, the need for admission to an inpatient rehabilitation unit (IPRU), complication rates and patient-reported outcome measures (PROMs),	NR
Alibayli et al. 2024 Turkey NR	Prospective study	National Surgical Quality Improvement Program (NSQIP)	145	40 (27.6%)	105 (72.4%)	67.58 ± 9.35	48 (33.1%)	42 (28.9%)	Modified frailty index (mFI)	30 days	Morbidity, mortality and postoperative outcome (postsurgical complications), duration of hospitalization, ICU admission, hospital readmission and reoperation.	NR
Teves et al. 2024 Argentina Jan 2016 – Dec 2019	Retrospective study	Electronic medical record (EMR)	448	NR	262 (58.5%)	82 (81-85)	NR	Group A = 39 (14.8%); Group B= 36 (19.5%)	Simple frailty score (SSF)	90- days	NR	NR
Yamaguchi et al. 2024 Japan Feb 2012 -Jan 2021	Retrospective study	Institutional database	434	77 (18%)	357 (82%)	76 (70–80)	NR	NR	Ageadjusted 5factor modified frailty index (aamFI5)	47 (24–71) mon-ths	Incidence of postoperative complications after BSTKA	NR
Zamanzadeh et al. 2024 USA Jan 2020 – Jul 2021	Prospective study	American College of Surgeons National Surgical Quality Improvement Program (ACS-NSQIP)	32, 069	13,310 (41.5%)	18,759 (58.5%)	66 (59 -74)	18762 (58.5%)	21,816 (68%)	Ageadjusted modified frailty index (aamFI); 5-item modified frailty index (mFI-5)	30- days	Primary outcome: any complication (>1); Secondary outcome: Clavien-Dindo IV Complication, 30- day Mortality, 30-day unplanned readmission, Nonhome Discharge	NR
Karumuri et al. 2023 India Jan 2020 – Dec 2021	Retrospective observational study	EMRs and institutional database	435	188 (43.21 %)	247 (56.78%)	70.03 ± 4.28	NR	49 (11.2%)	Frailty Index (FI)	1.2 Years	Change in FI (ΔFI)	Age, gender and BMI
Lakra et al. 2023 United States Jan 2017 – Nov 2019	Level III retrospective cohort study	Nationwide Readmission Database (NRD)	882, 479	NR	549,754 (62.29%)	18-74 Years	NR	62,321 (7.6%)	Hospital Frailty Risk Score (HFRS)	30- days	Primary outcome: 30- days readmission rate, 30- days complication rate and 30- days reoperation rate; Secondary outcome: length of the initial hospitalization and hospitalization cost	Adjusted but NR
Liu et al. 2023 China May 2015 – Oct 2015	Retrospective Observational study	Institution’s electronic medical system	811	192 (23.7)	619 (76.3)	67.86 ± 7.87	NR	38 (4.7%)	Clinical Frailty Scale (CFS), Modified Frailty Index (MFI)	NR	Primary outcome: Length of Stay (LOS) ; Secondary outcome: discharge location, 30-day readmission, inpatient complication, 30-day complication, 90-day complication, HD/ICU admission, 2-year reoperation and 90-day mortality	NR
Zalikha et al. 2023 United States 2006 -2015	Retrospective observational study	National Inpatient Sample (NIS)	7, 854, 890	Frail: 46, 781 (34.27 %); Non- frail: 3,018, 618 (39.11 %)	Frail: 89, 731 (65.73 %); Non- frail: 4,694, 180 (60. 82%)	67.3	NR	136,512	Modified Elixhauser Comorbidity Index (ECI)	NR	In-hospital postoperative outcomes	Adjusted but NR
Zamanzadeh et al. 2023 USA Jan 2015 – Dec 2019	Retrospective cohort study	American College of Surgeons National Surgical Quality Improvement Program (ACS-NSQIP)	271, 271	38.1	61.8	67 (61-73)	NR	NR	Ageadjusted modified frailty index (aamFI); 5-item modified frailty index (mFI-5)	NR	Primary outcome: any complication (>1); Secondary outcome: Clavien-Dindo IV Complication, 30- day Mortality, 30-day unplanned readmission, Nonhome Discharge	NR
Cook et al. 2022 UK Jan 1998 – Mar 2019	Retrospective cohort study	Clinical Practice Research Datalink (CPRD)	2,28, 930	NR	Knee cohort: 71, 169 (56.8) ; Hip cohort: 63,405 (61.2)	72.3 ± 7.2	125,367 (> 99.9 %)	Mild frailty: 55,845 (44.6) ; Moderate frailty: 22,056 (17.6) ; Severe frailty: 5,127 (4.1)	electronic Frailty Index (eFI)	90 - days	Impact of frailty on the risk of 30-, 60- and 90-day mortality following THA and TKA	Sex, 5-year age bands, quintile of IMD and year of surgery
Meyer et al. 2021 Germany Jan 2011 – Dec 2019	Level IV – retrospective cohort study	The department’s joint registry and the hospital information system	565	241 (42.6 %)	324 (57.3%)	69.1	234 (41.41 %)	NR	Hospital Frailty Risk Score (HFRS)	90- days	Primary outcome: Reoperation rate within first 90- days ; Secondary outcome: 90- days readmission rate and postoperative complications related to HFRS values	NR
Meyer et al. 2020 Germany Jan 2011 – Dec 2020	Level III– retrospective cohort study	The department’s joint registry and the hospital information system	8,250	3,500 (42.4%)	4,750 (57.6%)	84.2	3692 (44.7%)	NR	Hospital Frailty Risk Score (HFRS)	90- days	Primary outcome: Reoperation rate within first 90- days; Secondary outcome: 90- days readmission rate and postoperative complications related to HFRS values	Adjusted but NR
Schwartz et al. 2020 Georgia 2006 - 2018 and 2011 - 2018	Retrospective cohort study	American College of Surgeons National Surgical Quality Improvement Program (ACS-NSQIP)	179, 702	111,333 (62%)	68,369 (38%)	66.4 ± 9.6	142,756 (44.3%)	18.60%	modified frailty index (mFI)	NR	Incidence of complications	Adjusted but NR
Shin et al. 2016 USA 2005 – 2012	Retrospective cohort study	National Surgical Quality Improvement Program (NSQIP)	39, 807	THA= 44.4% ; TKA= 36.5%	THA= 55.6% ; TKA= 63.5%	67.1	25, 223 (63.36 %)	NR	modified frailty index (mFI)	NR	Clavien IV complications, Any complication, Hospital-acquired conditions and Mortality	Age≥75, BMI ≥ 40, Obesity class III, ASA class ≥4, Nonclean wound, mFI≥0.4

Most studies were retrospective cohort (n=11), complemented by four prospective studies.^25^,^28^,^32^,^38^ Additionally, two studies by Meyer et al. (2020, 2021) were designed as level III/IV cohort studies.^34^,^36^ Data were extracted from a variety of sources, including institutional electronic medical records (EMRs), national-level databases such as the American College of Surgeons National Surgical Quality Improvement Program (NSQIP), Nationwide Readmissions Database (NRD), National Inpatient Sample (NIS) and Clinical Practice Research Datalink (CPRD), as well as institutional joint registries. Geographically, most studies were conducted in developed countries—namely the United States, Germany, the United Kingdom and Australia—while three studies originated from emerging economies: India, Turkey and Argentina.

The sample sizes varied considerably across studies, ranging from 434 patients in the study by Yamaguchi et al.^26^ to 7,854,890 patients in the nationwide dataset analyzed by Zalikha et al.^29^ The average patient age across the studies ranged between 66 and 84 years, with female patients representing the majority in most cohorts. Notably, the reported prevalence of frailty varied widely, from as low as 4.7% in the cohort studied by Liu et al.^30^ to as high as 68% in Zamanzadeh et al.^25^

There was a heterogeneity in frailty assessment tools, such as the Hospital Frailty Risk Score (HFRS) used by O’Connor et al.,^24^ Meyer et al.,^34^,^36^ and Lakra et al.;^31^ the modified frailty index (mFI) and its age-adjusted versions by Liu et al.,^30^ Zamanzadeh et al.,^25^,^28^ Schwartz et al.,^35^ and Shin et al.^37^ the Clinical Frailty Scale (CFS) used by Wall et al.^23^ and Liu et al.^30^ and the electronic frailty index (eFI) applied in the UK-based study by Cook et al.^33^ Follow-up periods ranged from 30 days to nearly six years,^26^ with most studies focusing on 30- or 90-day outcomes.

Assessment of the methodological quality is summarized in Table-II. One study achieved the highest score of nine, indicating strong methodological rigor across all domains. Four studies received a score of eight, demonstrating a robust design with adequate comparability and thorough follow-up. The remaining 11 studies scored seven points, reflecting moderate to good quality.

**Table-II:** Quality of included studies using New castle – Ottawa Scale.

Study	Year	Selection				Comparability	Outcome			Total
		Representativeness of the exposed cohort	Selection of the nonexposed cohort	Ascertainment of exposure	Demonstration that outcome of interest	Basis of the design or analysis	Assessment of outcome	Follow- up long enough for outcomes	Ad- equate follow up	
O’Connor et al.	2025	0	1	1	1	1	1	1	1	7
Wall et al.	2025	0	1	1	1	1	1	1	1	7
Alibayli et al.	2024	0	1	1	1	1	1	1	1	7
Teves et al.	2024	1	1	0	1	1	1	1	1	7
Yamaguchi et al.	2024	1	1	1	1	2	1	1	1	9
Zamanzadeh et al.	2024	1	1	0	1	1	1	1	1	7
Karumuri et al.	2023	1	1	1	1	1	1	1	1	8
Lakra et al.	2023	1	1	1	1	1	1	1	1	8
Liu et al.	2023	1	1	1	1	1	1	1	1	8
Zalikha et al.	2023	0	1	1	1	1	1	1	1	7
Zamanzadeh et al.	2023	1	1	0	1	1	1	1	1	7
Cook et al.	2022	1	1	0	1	1	1	1	1	7
Meyer et al.	2021	1	1	0	1	1	1	1	1	7
Meyer et al.	2020	0	1	1	1	1	1	1	1	7
Schwartz et al.	2020	1	1	1	1	1	1	1	1	8
Shin et al.	2016	1	1	0	1	1	1	1	1	7

### Meta-analysis:

Twelve studies with 14,707,361 patients were included in this meta-analysis. The pooled analyses consistently demonstrated that frailty was significantly associated with adverse outcomes.

### Thirty day Mortality:

**Fig.2:**
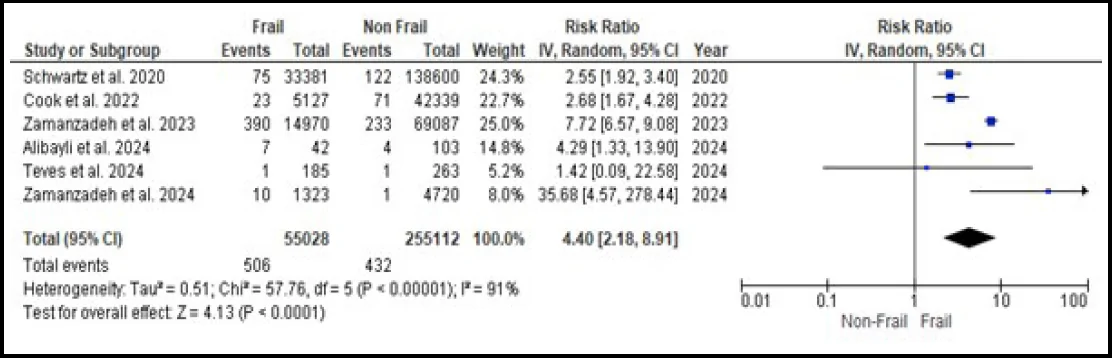
Pooled Risk ratio of 30-day mortality among frail patients undergoing TKA.

The pooled risk ratio (RR) for 30-day mortality among frail patients was 4.40 (95% CI: 2.18 to 8.91; p < 0.00001; I² = 91%), suggesting that frail patients have nearly a four-fold increased risk of mortality within 30 days post-TKA compared to non-frail patients (Fig.2).

### All complications:

**Fig.3:**
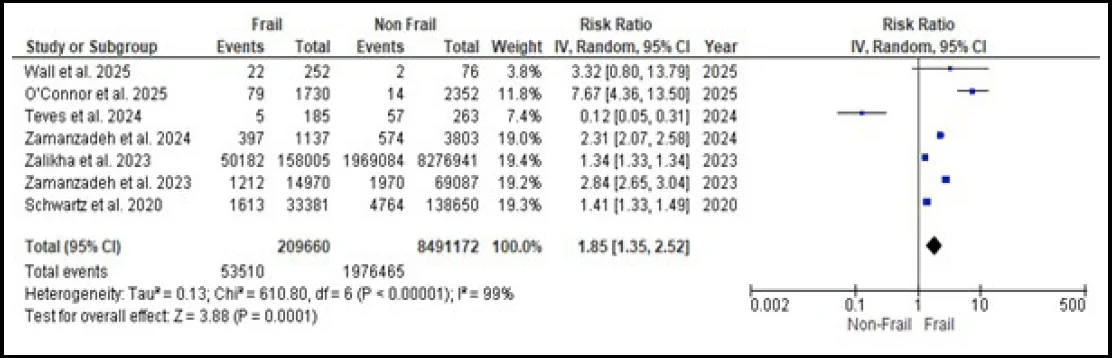
Pooled Risk ratio of All complications among frail patients undergoing TKA.

The pooled RR for all complications in frail patients was 1.85 (95% CI: 1.35 to 2.52; p= 0.0001; I² = 99%) (Fig.3). This robust association underscores frailty as a major predictor of postoperative complications following TKA.

### Thirty day Readmission and Reoperation:

Frailty was also associated with a significant increase in the risk of 30-day readmission (RR: 2.24; 95% CI: 1.74 to 2.87; p < 0.00001; I² = 97%) and 30-day reoperation (RR: 1.81; 95% CI: 1.48 to 2.22; p < 0.0001; I² = 82%).

### Surgical Site Infection:

The pooled RR for surgical site infections among frail patients was 1.56 (95% CI: 0.86 to 2.83; p = 0.14; I² = 93%).

### Adjusted Odds Ratio:

When adjusted for age and body mass index (BMI), frail patients still demonstrated a significantly increased risk for all complications (pooled OR: 2.20; 95% CI: 1.68 to 2.88; p < 0.00001; I² = 79%). The unadjusted odds ratio for all complications was similarly significant (OR: 2.89; 95% CI: 2.14 to 3.90; p < 0.00001; I² = 88%). The pooled adjusted odds ratio for 30-day readmission in frail patients was 1.77 (95% CI: 1.28 to 2.44; p = 0.0004; I² = 79%).

### Length of Hospital Stay:

Frailty was significantly associated with a prolonged length of hospital stay (mean difference: 0.86 days; 95% CI: 0.55 to 1.16; p < 0.00001; I² = 59%).

### Individual Postoperative Complications:

Pooled analyses of individual complications revealed that frailty significantly increased the risk of peri-prosthetic fracture, deep vein thrombosis, pulmonary embolism, myocardial infarction, septic shock, renal complications and the need for post-operative transfusion (Table-III).

**Table-III:** Pooled Risk -ratios of complications in Frail patients undergoing TKA.

Complication	No. of Studies	Sample Size	RR (95% CI)	p-value	I² (%)
Other Complications (Total)	12	14,707,361	1.85 [1.35, 2.52]	p < 0.00001	99%
Peri-prosthetic fracture	4	889,008	2.52 [1.95, 3.25]	p < 0.00001	0%
Delayed Wound Healing	4	842,473	2.09 [0.62, 7.01]	p = 0.23	77%
Deep Vein Thrombosis	6	8,489,652	1.17 [1.01, 1.35]	p = 0.04	55%
Pulmonary Embolism	7	1,036,310	1.55 [1.28, 1.88]	p < 0.0001	52%
Myocardial Infarction	5	1,212,360	4.48 [1.66, 12.07]	p = 0.003	91%
Septic Shock	4	244,660	5.74 [2.54, 12.97]	p < 0.0001	62%
Cerebro-vascular Disease	6	1,063,244	3.46 [0.67, 17.70]	p = 0.14	98%
Post-operative Delirium	4	842,473	5.81 [0.21, 159.62]	p = 0.30	99%
Renal Complication	5	1,063,611	5.49 [3.18, 9.45]	p < 0.00001	86%
Post-op Transfusion	5	1,213,443	2.31 [1.08, 4.96]	p = 0.03	99%

Conversely, non-significant associations were observed for delayed wound healing (RR = 2.09; 95% CI: 0.62 to 7.01; p = 0.23; I² = 77%), cerebro-vascular disease (RR = 3.46; 95% CI: 0.67 to 17.70; p = 0.14; I² = 98%) and post-operative delirium (RR = 5.81; 95% CI: 0.21 to 159.62; p = 0.30; I² = 99%).

The certainty of evidence assessed using the GRADE approach was low and very low for most major outcomes, including mortality, overall complications, readmission, reoperation, and surgical site infection, mainly downgraded due to heterogeneity among studies. Low to very low certainty was observed for outcomes such as myocardial infarction, renal complications, delirium and delayed wound healing because of inconsistency and imprecision. Moderate rate of certainty was provided for Deep vein thrombosis and pulmonary embolism events (Table-IV).

**Table-IV:** GRADE approach for level of certainty for all outcomes

Outcome	No. of Studies	Participants	Effect Estimate	Heterogeneity (I²)	Certainty of Evidence (GRADE)	Visual Rating	Reason for Downgrading
30-day mortality	6	>1 million	RR 4.40 (95% CI: 2.18–8.91)	91%	Low	++◯◯	Downgraded for substantial heterogeneity
Overall postoperative complications	12	14,707,361	RR 1.85 (95% CI: 1.35–2.52)	99%	Very Low	+◯◯◯	Downgraded for substantial heterogeneity
30-day readmission	6	>1 million	RR 2.24 (95% CI: 1.74–2.87)	97%	Very Low	+◯◯◯	Downgraded for moderate heterogeneity
30-day reoperation	5	>1 million	RR 1.81 (95% CI: 1.48–2.22)	82%	Low	++◯◯	Downgraded for inconsistency
Surgical site infection	5	>1 million	RR 1.56 (95% CI: 0.86–2.83)	93%	Low	++◯◯	Downgraded for moderate heterogeneity
Length of hospital stay	7	>1 million	MD 0.86 days (95% CI: 0.55–1.16)	59%	Low	++◯◯	Downgraded for inconsistency and observational study design
Deep vein thrombosis	6	8,489,652	RR 1.17 (95% CI: 1.01–1.35)	55%	Moderate	+++◯	Downgraded for moderate heterogeneity
Pulmonary embolism	7	1,036,310	RR 1.55 (95% CI: 1.28–1.88)	52%	Moderate	+++◯	Downgraded for moderate heterogeneity
Myocardial infarction	5	1,212,360	RR 4.48 (95% CI: 1.66–12.07)	91%	Low	++◯◯	Downgraded for serious inconsistency and imprecision
Renal complications	5	1,063,611	RR 5.49 (95% CI: 3.18–9.45)	86%	Low	++◯◯	Downgraded for substantial heterogeneity
Septic shock	4	244,660	RR 5.74 (95% CI: 2.54–12.97)	62%	Low	++◯◯	Downgraded for inconsistency and imprecision
Postoperative delirium	4	842,473	RR 5.81 (95% CI: 0.21–159.62)	99%	Very Low	+◯◯◯	Downgraded for extreme heterogeneity and imprecision
Cerebrovascular disease	6	1,063,244	RR 3.46 (95% CI: 0.67–17.70)	98%	Very Low	+◯◯◯	Downgraded for extreme heterogeneity and imprecision
Delayed wound healing	4	842,473	RR 2.09 (95% CI: 0.62–7.01)	77%T	Very Low	+◯◯◯	Downgraded for inconsistency and imprecision

## DISCUSSION

The aim of this study was to assess if frailty could be a predictor of postoperative complications and mortality in patients undergoing TKA. By combining data from over 14 million patients from sixteen studies, the results showed that frailty can be a robust and independent predictor of poor outcomes after TKA.

An important outcome noted in our analysis is that frailty was linked with nearly four-fold increase in the risk of 30-day mortality. This is consistent with previous research. A study by Zamanzadeh et al. has shown that frailty significantly elevates the risk of mortality in TKA patients, even after adjustment for age.^28^ It is possible that the impact of frailty on mortality on mortality is due to the diminished physiological reserves in such patients which causes impairment of cardiovascular function and reduces the ability to provide an adequate stress response in the perioperative period.^39^

Other than mortality, our analysis also showed that frail patients had a significantly higher risk of overall postoperative complications. These results are similar to the findings of most of the included studies. Lakra et al. showed that frailty led to increased risk of complications, longer hospitalization periods and increased healthcare costs following TKA.^31^ Likewise, Garcia et al. noted that a higher modified frailty index was directly associated with higher risk of 30-day postoperative complications in patients undergoing simultaneous bilateral TKA.^20^ Their data also showed that frailty was linked with higher risks of peri-prosthetic fractures, pulmonary embolism and myocardial infarction. This suggests that frail patients have a number of musculoskeletal, cardiovascular and thrombotic vulnerabilities leading to higher risk of various complications.

We also found a higher 30-day readmission and reoperation rates in frail patients. This suggests that the effect of frailty may be prolonged and extend into the immediate postoperative period. Previous systematic reviews^14^,^15^ have similarly show that frail patients have significant postoperative functional decline. The same reviews have also shown an association between frailty and delayed wound healing and reduced participation in rehabilitation programs, predisposing them to readmission for several complications like joint stiffness, infections and thromboembolic events. Our analysis also noted approximately 1.6-fold higher risk of surgical site infections in frail patients. Wilson et al. have shown that in THA patients, the coexistence of frailty and hypoalbuminemia, which is a surrogate for malnutrition, increases the risk of infections and other complications.^39^ This combined effect of frailty and malnutrition suggests that nutritional optimization may help reduce the risk of infections in frail patients. Indeed, this area needs further robust research. Length of hospital stay was significantly prolonged in frail patients, with a mean difference of 0.86 days. Prolonged hospitalization increases utilization of healthcare resources, increasing costs and also exposes patients to higher risk of hospital-associated complications like nosocomial infections.

There are several possible pathways by which frailty can adversely affects postoperative outcomes. Firstly, frail individuals have chronic low-grade inflammation, sarcopenia, reduced immune function and poor nutritional status. A combination of these factors reduces physiological reserve and the ability of the individual to respond to surgical stress. Moreover, these bodily changes increase the risk of infections, prolong wound healing, and increase the risk of thromboembolic events and cardiovascular complications. Secondly, reduced muscle strength and functional impairment may affect the patients ability for postoperative mobilization and rehabilitation. This leads to increased recovery time while simultaneously increasing the risk of readmission and mortality.^40^ Thirdly, cognitive impairment in frail patients may also hinder postoperative adherence to rehabilitation protocols, thereby increasing the risk of adverse events.^41^–^43^

Similar impact of frailty has also been reported for other orthopaedic procedures, like THA and spine surgery. Zalikha et al.^29^ have shown that frail patients undergoing both THA and TKA experienced significantly higher rates of in-hospital complications as compared to non-frail patients. Similarly, Traven et al.^44^ noted that frailty was a strong predictor of readmission and mortality following revision hip and knee arthroplasty. In spine surgery, frailty has been shown to increase the risk of postoperative delirium, infection and pulmonary complications.^45^ These findings indicate that frailty is general risk factor that affects outcomes across a wide range of orthopaedic interventions. Early identification and and tailored perioperative management may therefore help in reducing adverse events in a number of musculoskeletal surgeries. Our results are also comparable to the previous meta-analyses by Wu et al.^16^ and Lan et al.^17^ However, these reviews focused specifically on surgical site infections and venous thromboembolic events after THA/TKA and did not include a comprehensive assessment of all complications. Our analysis additionally assessed mortality, readmission, reoperation and hospital stay thereby improving and expanding the evidence.

A major limitation of our analysis was the heterogeneity in frailty assessment tools used by the studies. These tools varied in their structure, scoring criteria and the physiological domains. Some used comorbidity burden and functional status while other used cognitive and social vulnerability. For example, the mFI predominantly relies on comorbid conditions and has it has limitations of underestimating functional decline. On the other hand, the CFS is a more detailed tool, but has bias due to subjectivity. This inconsistency likely resulted in vast difference in frailty prevalence (4.7% to 68%) in the included studies and may have skewed the results. Future research should focus on standardizing frailty assessment tools to allow comparisons and pooling of data across multiple studies. Additionally, interventional studies examining the impact of preoperative optimization strategies, like nutrition support, physical prehabilitation and comprehensive geriatric assessments, on reducing frailty-related risks in TKA patients are needed.

### Strengths

The findings were obtained from 16 studies leading to a large sample size. We comprehensive evaluated several clinically relevant outcomes unlike priors reviews.

### Limitations:

First, most included studies were retrospective. We cannot completely exclude the possibility of coding errors and unmeasured confounding due to several missed clinical variables. Second, there was heterogeneity in the frailty assessment tools used by the studies leading to potential measurement bias. Such variations can prevent uniform interpretation of frailty severity. Third, while the adjusted analyses were conducted where possible, not all studies reported adjusted effect estimates. The covariates included in the studies varied thereby potentially affecting the validity of results. Finally, most included studies were conducted in high-income countries with advanced healthcare infrastructure. Hence, the findings may not be generalizable to low-resource settings.

### Clinical implications:

The present results show that identifying frail patients preoperatively can improve risk stratification in TKA patients. Such patients may benefit from added nutritional support and rehabilitation. Frailty screening may also help clinicians develop individualized patient counselling and discharge plans thereby reducing postoperative complications, mortality and healthcare burden in TKA patients.

## CONCLUSION

Frailty can be a significant and independent predictor of postoperative complications, mortality and prolonged hospital stays in patients undergoing TKA. However, high heterogeneity of evidence especially for frailty assessment tools is an important limitation preventing strong conclusions. Further robust studies using standardized criteria are needed for better evidence.

### Author’s contributions:

**JB:** Literature search, study design and manuscript writing.

**JW, YH and WR:** Data collection, data analysis and interpretation. Critical Review.

**JB:** Manuscript revision and validation and is responsible for the integrity of the study.

All authors have read and approved the final manuscript.
